# The clinical and genetic aspects of six individuals with *GH1* variants and isolated growth hormone deficiency type II

**DOI:** 10.3389/fendo.2024.1363050

**Published:** 2024-10-07

**Authors:** Xiaozhen Huang, Hong Chen, Huakun Shangguan, Wenyong Wu, Zhuanzhuan Ai, Zhifeng Chen, Ruimin Chen

**Affiliations:** Department of Endocrinology, Genetics and Metabolism, Fuzhou Children’s Hospital of Fujian Medical University, Fuzhou, Fujian, ;China

**Keywords:** *GH1* gene, growth hormone deficiency type II, short stature, pulmonary hypertension, rhGH treatment

## Abstract

**Background:**

Isolated growth hormone deficiency type II (IGHD II) is an autosomal dominant disorder characterized by a *GH1* gene variant resulting in a significant reduction in growth hormone (GH) secretion and a subsequent decrease of plasma insulin-like growth factor 1 (IGF-1) levels and eventual growth impairment.

**Objective:**

This study aimed to identify causative variants in six Chinese families with IGHD II, exploring both clinical and genetic characteristics.

**Methods:**

Detailed clinical data, including clinical presentations, physical charateristics, medical and family histories, as well as genetic test results, were systematically examined.

**Results:**

Six children, comprising four males and two females, with a mean age of 4.64 ± 1.15 years, exhibited short stature with a mean height of -3.95 ± 1.41 SDS. Four of them had a family history of short stature, while one patient presented with pulmonary hypertension. All children demonstrated GH deficiency in growth hormone stimulation tests (mean peak GH value: 2.83 ± 2.46 ng/mL). Exome sequencing for the six patients and targeted gene sequencing for their family members revealed heterozygous variants in the *GH1* gene, including Exon2-5del, c.334T>C, c.291 + 1G>A, c.291 + 2T>A, 1.5 kb deletion, and 1.7 kb deletion, with four variants being novel. Four patients underwent human recombinant growth hormone (rhGH) replacement therapy, initiating treatment at a mean age of 4.6 ± 0.7 years. The mean height increase in patients was 1.21 ± 0.3 SDS in the first six months of treatment and 1.79 ± 0.15 SDS in the first year.

**Conclusion:**

Our findings contribute to expanding the genotypic and phenotypic spectra of individuals with IGHD II.

## 1 Introduction

Human height is influenced by a complex interplay of genetic, hormonal, nutritional, and environmental factors. Growth hormone (GH), a polypeptide hormone synthesized and secreted in the anterior pituitary gland, plays a crucial role in regulating somatogenic and metabolic processes in humans (1–3). Growth hormone deficiency (GHD) is a condition characterized by inadequate secretion or structural abnormalities of GH, leading to impaired growth (4). GHD can be categorized into isolated growth hormone deficiency (IGHD) and combined pituitary hormone deficiency, depending on the presence of deficiencies in other pituitary hormones.

IGHD is a prevalent pituitary hormone deficiency, with an incidence ranging from 1/4000 to 1/10000, predominantly occurring sporadically. Familial cases constitute 3% to 30% of all cases (5). Variations in the *GH1* gene are a significant contributor to IGHD, classified into two genetic patterns: autosomal recessive inheritance (IGHD types IA and IB) and autosomal dominant (IGHD II) (6). IGHD II, the most common genetic form, is characterized by short stature and delayed bone age, accompanied by laboratory findings of low but detectable serum GH and reduced insulin-like growth factor 1 (IGF-1) levels. Magnetic resonance imaging (MRI) typically reveals a normal or hypoplastic anterior pituitary. Treatment with human recombinant growth hormone (rhGH) has proven effective in improving height in IGHD II patients (7, 8). However, understanding genotype-phenotype correlations can better help to assess the efficacy and safety of rhGH treatment, therefore, we analyzed the clinical manifestations, laboratory examinations and genetic testing results, as well as the growth responses of rhGH treatment on six patients carrying heterozygous variant in *GH1* gene.

## 2 Patients and methods

### 2.1 Subjects

Fujian Medical University between June 2016 and February 2024 were identified by Whole Exon Sequencing (WES). This study, conducted in accordance with the Declaration of Helsinki, received approval from the Ethics Committee of Fuzhou Children’s Hospital of Fujian Medical University (approval number 202310). Prior to participation, written informed consent was obtained from the parents of the patients.

Inclusion criteria: (1) The measured height of the children (measured body length of children under 3 years old) was lower than the mean third percentile (-2 SDS) of the population of the same race, age and sex; (2) WES testing; (3) Growth hormone stimulation tests were performed. Patients with a peak GH value < 10 ng/mL were classified as GHD (9); (4) The patient had complete medical records.

Exclusion criteria: (1) hypothalamic-pituitary dysfunction caused by organic lesions such as intracranial tumors and cranial trauma; (2) congenital hypothyroidism; (3) chromosome diseases; (4) inherited metabolic bone diseases, such as congenital chondrodysplasia and mucopolysaccharidosis; (5) other chronic diseases, such as congenital heart disease, chronic hepatitis, henoch-schonlein purpura nephritis, and inflammatory bowel disease.

### 2.2 Clinical evaluations

Detailed clinical data, including age, gender, chief complaint, physical chararteristics, as well as medical and family histories were systematically collected. Growth hormone stimulation tests utilized L-dopa (10 mg/kg) and arginine (0.50g/kg). Serum levels of thyroid hormones, GH, IGF-1, and IGFBP3 were measured using chemiluminescent immunoassay. Bone age was determined using Siemens’ direct digital radiography machine and Agfa’s computer X-ray system, with assessment conducted via the Tanner-Whitehouse III (TW3) method (10). The standard deviation scoreof IGF-1 was calculated relative to levels in healthy children of the same age and gender (11). Throughout the rhGH treatment, patients underwent follow-up examinations every three to six months.

### 2.3 Genetic examination

A total of 2 mL of venous blood collected in EDTA anticoagulant was obtained from the proband and their family members. Genomic DNA extraction from peripheral blood followed standard procedures, and whole exome sequencing was conducted using established protocols. The *GH1* gene was referenced against the National Center for Biotechnology Information (NCBI) entry NG_011676.1 (NM_000515.5). Pathogenicity analysis of genetic variants was performed in accordance with guidelines from the American College of Medical Genetics and Genomics and the Association for Molecular Pathology (12, 13). Validation and segregation analysis of candidate gene variants were carried out through Sanger sequencing.

### 2.4 Conservation and pathogenicity analysis of the GH^W112R^ variant

Conservation of amino acid substitution positions across species was assessed using ClustalX 1.83 software (14). The pathogenicity of the GHW112R variant was predicted using the PREDICTSNP web server (https://loschmidt.chemi.muni.cz/predictsnp1/), which integrates multiple predictors (PredictSNP, MMAP, PhD-SNP, polyphen1, polyphen2, SIFT, and SNAP) with a confidence score (15).

### 2.5 Homology modeling and structural analysis of human GH

For the modeling of human GH, the crystal structure model of the wild-type GH (amino acid: 27-217) (PDB entry: 1HUW) was used. Mutant proteins were constructed using the Iterative Threading ASSEmbly Refinement (I-TASSER) server (https://seq2fun.dcmb.med.umich.edu/I-TASSER/) (16). The HOPE server (https://www3.cmbi.umcn.nl/hope/) analyzed the effect of variation on the three-dimensional structure of GH (17). SWISS-MODEL online software (https://swissmodel.expasy.org/) compared the three-dimensional structural alterations between wild-type and mutant GH. The DynaMut server (http://biosig.unimelb.edu.au/dynamut/) was employed to predict the interaction between amino acid residues and analyze protein stability and flexibility. Finally, PyMOL 2.5 was used to visualize the modeled proteins.

### 2.6 Literature review

IGHD II patients were additionally identified through a comprehensive search of the PubMed database for published clinical cases utilizing keywords such as “isolated growth hormone deficiency”, “*GH1* gene”, or/and “growth hormone”.

### 2.7 Statistical analysis

Based on the distribution of variables, we presented them in this study as mean ± standard deviation for normally distributed variables, or median (min-max) for skewed distributions. Categorical data are typically displayed in the form of both numerical counts (n) and corresponding percentages (%). The One-way Analysis of Variance (ANOVA) test was employed to assess the differences in height SDS and diagnosis age among patients with various GH1 heterozygous variants, utilizing the statistical software SPSS.27. The chart was created using GraphPad Prism 10.1.2 software. * indicates *p* < 0.05, ** indicate *p* < 0.01, *** indicate *p* < 0.001, and **** indicate *p* < 0.0001.

## 3 Results

### 3.1 Clinical evaluations

792 unrelated short stature patients ascertained in Fujian Province, China, were genetically evaluated by WES–based test. We identified three novel pathogenic variants of the *GH1* gene, two recurrent pathogenic variants, and one novel likely pathogenic variant in this cohort. The variants in patients 2, 4, 5, and 6 were inherited from their affected parents, whereas the variants in patients 1 and 3 were *de novo*.

#### 3.1.1 Patient 1

The patient 1 was a 3.8-year-old boy who was delivered by cesarean section at full term. His birth weight was 3100g, and birth length 48cm. He has a proportionate short stature (height 92.3cm, -2.8SD) without additional atypical physical features. The BA was 2.25 years using the TW3 method. The GH stimulation test results included a GH level of 3.22ng/mL (normal: ≥10ng/mL), and an IGF-1 level of 46.3ng/mL (**Table 1**). The levels of thyroid hormone, calcium, phosphate, and parathyroid hormone were all within normal ranges. The pituitary Magnetic resonance imaging (MRI) showed no abnormality. The heights of the father and mother were 165cm (-1.2SDS), 160cm (-0.1SDS), respectively.

**Table 1:** Clinical data of patients.

Items	Patient 1	Patient 2	Patient 3	Patient 4	Patient 5	Patient 6	
Gender	Male	Male	Male	Male	Female	Female	
Birth weight (kg)	3.10	3.00	3.50	3.00	2.80	3.40	
Birth height (cm)	48	50	53	48	50	49	
Age at diagnosis	3.83	5.50	4.08	4.33	3.33	6.75	
Height (cm)	92.3	91.1	87.1	82.3	86.8	110.7	
HSDS	-2.80	-5.24	-4.51	-6.05	-2.97	-2.15	
Weight (kg)	12.2	11.0	13.5	9.3	11.1	15.7	
BMI (kg/m^2^)	14.3	13.3	17.8	13.7	14.7	12.8	
Bone age (years)	2.25	3.17	3.08	3.42	2.75	6.33	
Additional clinical manifestations	N	N	N	prominent, bossing forehead, long eyelashes, sparse eyebrows at the lateral third, short philtrum, and thin lips. pulmonary hypertension, micropenis, cryptorchidism	significant hair growth on temples and back, a depressed nasal bridge, long snout, and short philtrum	N	
MRI	N	N	N	a small pituitary gland and abnormal signals behind the genu of the corpus callosum, possibly a cyst	a Rathke’s cleft cyst	N	
Blood hormonal characteristics							Normal value
Peak growth hormone	3.22	2.05	0.29	0.15	5.59	5.70	≥10ng/mL
IGF-1 (pg/mL)	46.3	25	<25	<15	38.2	68.2	
IGF-1 SDS	-1.53	-2.51	<-2.39	<-2.76	-2.86	-2.30	
IGFBP3 (mg/L)	ND	ND	0.77	<0.5	3.67	2.15	0.7~10mg/L

SDS, standard deviation score; HSDS, height standard deviation score; IGF-1, insulin-like growth factor-1; IGFBP3, insulin-like growth factor binding protein-3; ND, not done; N, normal.

#### 3.1.2 Patient 2

The patient 2 was a 5.5-year-old boy who was delivered at full term by natural birth. His birth weight was 3000g, and birth length 50cm. His latest height and weight were 91.1cm (-5.24SDS) and 11.0kg (-2.57SDS), respectively. He showed a thin body habitus and proportional short stature without additional physical features. His GH stimulation tests revealed a GH deficiency. **Table 1** shows the levels of hormones. The BA was 3.17 years. His father’s and mother’s heights were 152cm (-3.39SDS) and 152cm (-1.59SDS) respectively.

#### 3.1.3 Patient 3

The patient 3, a 4.1-year-old boy who was delivered by cesarean section at full term. His birth weight was 3500g, and birth length 53cm. His latest height and weight were 87.1cm (-4.51SDS) and 13.5kg (+1.17SDS), respectively. His GH stimulation test and IGF-1 level test results were low (**Table 1**). The BA was 3.08 years. His mother and father’s height were 156 (-0.85SDS) and 177cm (+1.4 SDS), respectively.

#### 3.1.4 Patient 4

The patient 4 was a 4.3-year-old boy, who was delivered by cesarean section at full term. His birth weight was 3000g, and birth length 48cm. His latest height and weight were 82.3cm (-6.05SDS) and 9.3kg (-2.57SDS), respectively. He was proportional short stature and wasting (thin-for-height) with characteristic face (bossing forehead, long eyelashes, sparse eyebrows at the lateral third, short philtrum and thin lips). He had a short penis. A grade 2 pansystolic murmur was heard at the 4th intercostal space along the left sternal border. Color Doppler echocardiography revealed moderate tricuspid regurgitation and pulmonary hypertension (systolic pulmonary artery pressure was 40mmHg). He underwent surgery for right-sided cryptorchidism at the age of two. His GH stimulation test and IGF-1 level were low (**Table 1**). The BA was 3.42 years. MRI revealed a small pituitary gland and abnormal signals behind the genu of the corpus callosum, possibly a cyst (**Supplementary Figure 1B**). His mother and father’s height are 130 (-5.67SDS) and 138cm (-5.69 SDS), respectively. His mother also had characteristic face (sparse eyebrows at the lateral third, thin lips and micromandible). His maternal grandmother was also short with a height of 118cm (-7.89SDS).

#### 3.1.5 Patient 5

The patient 5 was a 3.3-year-old girl who was delivered by cesarean section at full term. Her birth weight was 2800g, and birth length 50cm. Her latest height and weight were 86.8cm (-2.97SDS) and 11kg (-1.13SDS), respectively. She has vigorous hair on her temples and back. Other features include a depressed nasal bridge, long snout and short philtrum. Blood hormone levels was listed in **Table 1**. The BA was 2.75 years. MRI of the pituitary showed a Rathke’s cleft cyst (**Supplementary Figure 1B**). Her mother’s heigh was 155cm (-1.0SDS) and had the typical facial features of an adult with GHD such as a depressed nasal bridge and long snout. Her father’s height was172cm (-0.1SDS). Her maternal grandmother was also short with a height of 145cm (-2.89SDS).

#### 3.1.6 Patient 6

The patient 6 was a 6.75-year-old girl who was delivered by spontaneous labor at full term. Her birth weight was 3400g, and birth length 49cm. Her latest height and weight were 110.7cm (-2.15SDS) and 15.7kg (-1.83SDS), respectively. **Table 1** shows the levels of blood hormones. The BA was 6.33 years. Her mother and father’s height are 154 (-1.2SDS) and 159cm (-2.24SDS), respectively.”

### 3.2 rhGH treatment and follow-up

**Figure 1:**
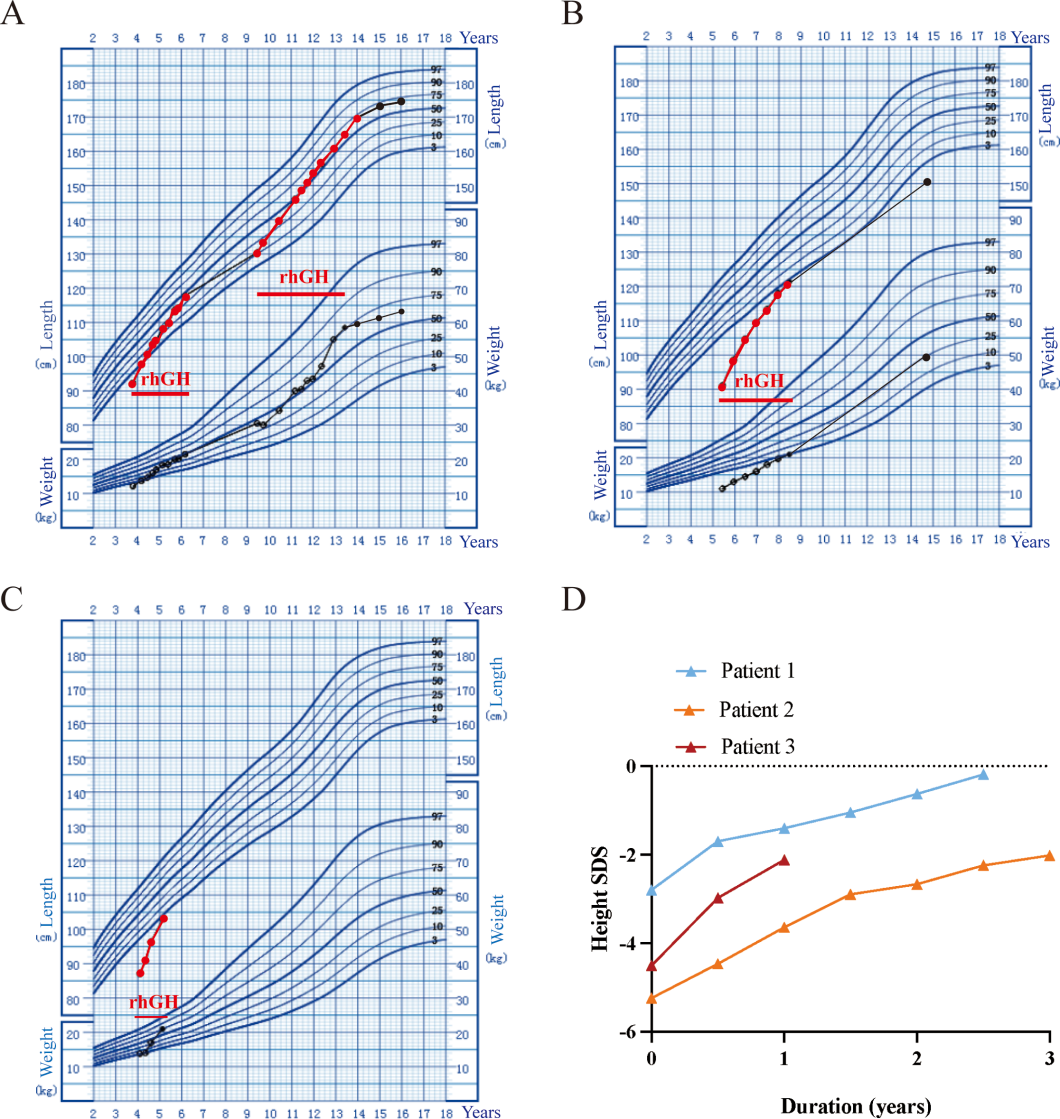
Growth chart of the three patients with heterozygous *GH1* variants with the rhGH therapies. Height and weight standardized growth charts for Chinese children and adolescents aged 0 to 18 years. The dots represent records. **(A)** Patient 1 height and weight trends during rhGH treatment. **(B)** Patient 2 height and weight trends during rhGH treatment. **(C)** Patient 3 height and weight trends during rhGH treatment. **(D)** rhGH treatment response in the three patients with IGHD II.

Four patients (Patients 1-4) underwent rhGH treatment at a dosage of 0.10~0.16 IU/kg/d (**Figure 1**; **Table 2**). The mean age at the initiation of treatment was 4.5 ± 0.7 years. In the initial year of treatment, the height standard deviation score (ΔHtSDS) increased by 1.79 ± 0.15 SDS. Patient 1, who commenced rhGH treatment at 3.8 years old, experienced a height increase to -0.19 SDS (ΔHtSDS: 2.61 SDS) after 2.3 years of treatment. Subsequently, he suspended rhGH treatment for 3.25 years, returning at 9.4 years of age with a height of 130.4cm (-1.12 SD). To achieve a more favorable height, he resumed rhGH treatment for 4.5 years, reaching a height of 169.5cm (+0.50 SDS) at 13.9 years, after which the treatment was discontinued. At the last follow-up at 16 years, the child measured 175.0cm (+0.54 SDS), surpassing the genetic target height of 169.5cm. Patient 2, treated with rhGH at 5.5 years, initially measured 91.1cm (-5.24 SDS) and achieved a height of -2.02 SDS (ΔHtSDS: 3.22 SDS) after 3 years of continuous treatment. Patient 3, treated at 4.1 years, started at 87.1cm (-4.51 SDS) and reached -2.12 SDS (ΔHtSDS: 2.37 SDS) after 1 year of treatment. Patient 4, treated at 4.9 years, started at 84.5cm (-6.09 SDS) and reached 94.5cm (-4.65 SDS, ΔHtSDS: 1.44 SD) after 6 months of treatment. Patient 5 and 6 had not received GH treatment due to economic reasons. Regular follow-ups during treatment indicated normal levels of blood and urine parameters, calcium and phosphorus metabolism, blood glucose, blood lipids, liver and kidney functions, and IGF-1 within the normal range. Detailed clinical information is provided in **Table 2**.

**Table 2:** Patients’ response to growth hormone therapy.

	Age (years)	Duration of GH treatment (years)	Dose of rhGH (IU/kg/d)	Height (cm)	HSDS	IGF-1 (pg/mL)	IGFBP3 (mg/L)
Patient 1	3.8	0	0.10	92.3	-2.80	46.3	ND
	4.3	0.5	0.10	100.5	-1.70	198	ND
	4.8	1	0.10	104.7	-1.40	144	ND
	5.3	1.5	0.10	109.8	-1.05	107	ND
	5.8	2	0.10	114.3	-0.63	213	ND
	6.1	2.3	0.11	117.8	-0.19	ND	ND
	9.4	Suspension of treatment for 3.25 years	0.10	130.4	-1.12	116.0	3.15
	10.4	3.3	0.11	139.7	-0.40	225.0	5.21
	11.4	4.3	0.13	148.6	-0.10	290.0	4.65
	12.4	5.3	0.16	156.3	+0.17	431.0	6.44
	13.4	6.3	0.16	165.0	+0.42	719.0	6.19
	13.9	6.8	0.16	169.5	+0.50	ND	ND
Patient 2	5.5	0	0.10	91.1	-5.24	25	ND
	6.0	0.5	0.10	98.6	-4.47	62.3	ND
	6.5	1	0.10	104.7	-3.64	65.2	ND
	7.0	1.5	0.11	109.5	-2.90	76.8	2.26
	7.5	2	0.11	113.3	-2.67	111	2.05
	8.0	2.5	0.13	118	-2.24	166	3.22
	8.5	3	0.14	121.4	-2.02	217	3.91
Patient 3	4.1	0	0.10	87.1	-4.51	<25	0.77
	4.6	0.5	0.10	96.4	-2.98	267	4.56
	5.1	1	0.10	102.8	-2.12	211	3.86
Patient 4	4.9	0	0.09	84.5	-6.09	<15	<0.5
	5.4	0.5	0.13	94.5	-4.65	133	4.37

HSDS, height standard deviation score; IGF-1, insulin-like growth factor-1; IGFBP3, insulin-like growth factor binding protein-3; ND, not done.

### 3.3 Genetic diagnosis

**Figure 2:**
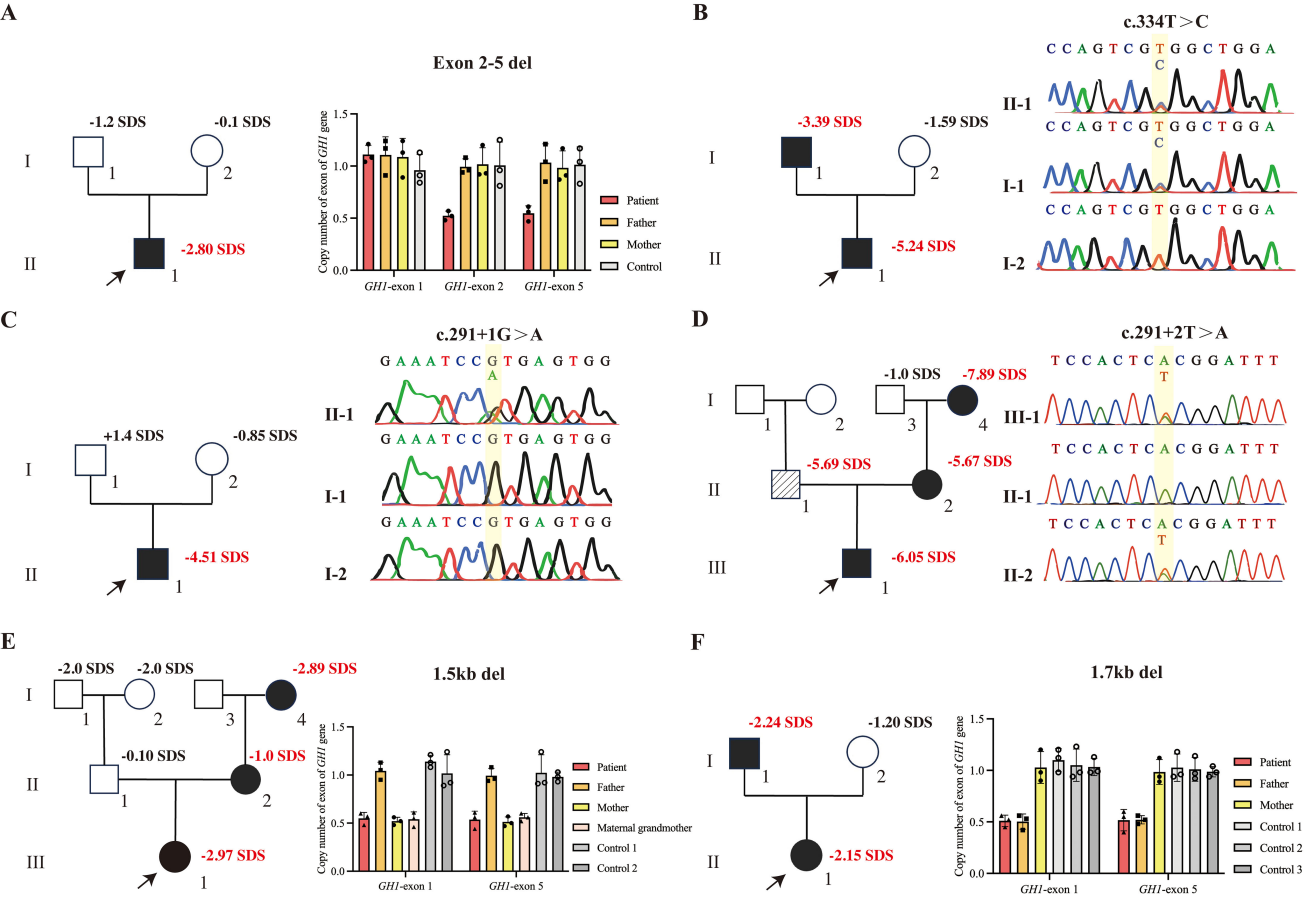
*GH1* gene variants in the patients and their family members. Squares represent males, and circles represent females. The proband is indicated by an arrow. Black-filled symbols indicate subjects with *GH1* gene variants. The oblique line filled-in symbol represents affected individuals carrying homozygous variants of the GHRHR gene. Bar chart demonstrating the copy number of *GH1*. **(A)** qPCR confirmed the heterozygous deletion (*GH1* gene exon 2-5del) in patient 1, neither of whose parents carried exon 2-5del. **(B)** Sanger sequencing chromatograms show that patient 2 carries the heterozygous c.334T>C variant, which was inherited from his father. **(C)** Sanger sequencing chromatograms show that patient 3 carries the heterozygous c.291 + 1G>A variant. The parents do not carry the variant. **(D)** Sanger sequencing chromatograms show that patient 4, his mother, and maternal grandmother carry the heterozygous c.291 + 2T>A variant of *GH1* gene. His father carried a homozygous variant of *GHRHR* gene (c.659T>C;p.Leu220Pro). **(E)** qPCR confirmed the heterozygous deletion of the *GH1* gene in patient 5 and her mother. **(F)** qPCR confirmed the heterozygous deletion of the *GH1* gene in patient 6 and her father.

A total of six *GH1* gene pathogenic/likely pathogenic variants was detected, thus, the overall prevalence of pathogenic *GH1* variants in this cohort was 0.76% (6/792). Among them, four variants were novel. Exome sequencing and quantitative PCR revealed a heterozygous deletion of exons 2-5 in the *GH1* gene in Patient 1 (**Figure 2A**). The variant was determined to be *de novo*, as it was not present in either parent. Sanger sequencing confirmed that Patient 2 carried a heterozygous missense variant of the *GH1* gene (c.334T>C, p.Trp112Arg), inherited from the affected father (**Figure 2B**). Patient 3 and Patient 4 carried heterozygous splicing site variants (c.291 + 1G>A, p.Glu58Asnfs*120 and c.291 + 2T>A, p.Glu58Asnfs*120), respectively. The variant in Patient 3 was *de novo* and the variant in Patient 4 was inherited from affected mother (**Figures 2C, D**). Additionally, the maternal grandmother of Patient 4 also carried the variant. Interestingly, the patient 4’s father carried a homozygous variant of *GHRHR* gene (c.659T>C;p.Leu220Pro), patient 4 was a heterozygous carrier. According to the ACMG guideline, it is a variants of unknown significance (PP3+PM3_Supporting+PM2). Homozygous variants in the *GHRHR* gene lead to isolated growth hormone deficiency type IV (MIM#618157), which may be the reason why the father of patient 4 does not carry the *GH1* gene mutation but has severe short stature clinical manifestations. Patient 5 carried a large fragment heterozygous deletion of approximately 1.5 kb, containing only the *GH1* gene (ex.1_5del), inherited from her mother, and her maternal grandmother (145cm, -3SDS) also carried the same heterozygous deletion (**Figure 2E**). Furthermore, we identified an approximately 1.7 kb heterozygous deletion in patient 6, encompassing only the *GH1* gene (ex.1_5del). Her father carried the same deficiency (**Figure 2F**). In summary, we reported six variants of the *GH1* gene, four were novel (exon2-5del, c.334T>C, 1.5 kb deletion of *GH1*, and 1.7 kb deletion of *GH1*) (**Table 3**).

**Table 3:** Genotypes of six patients.

	Variation (nucleotide)	Variation (protein)	Region	Genotype	Source of variation	ACMG criteria	
						Pathogenicity Evidence	Score
patient 1	Exon 2-5 del	NA	Exon 2-5	Heterozygous	*de novo*	NA	P
patient 2	c.334T>C	p.Trp112Arg	Exon 3	Heterozygous	paternal	PM1+PM2+PP3+PP4	LP
patient 3	c.291 + 1G>A	p.Glu58Asnfs*120	splice site	Heterozygous	*de novo*	PVS1+PM1+PP3+PP4	P
patient 4	c.291 + 2T>A	p.Glu58Asnfs*120	splice site	Heterozygous	maternal	PVS1_Strong+PM2+PP4	P
patient 5	1.5kb del	NA	Exon 1-5	Heterozygous	maternal	NA	P
patient 6	1.7kb del	NA	Exon 1-5	Heterozygous	paternal	NA	P

ACMG, American College of Medical Genetics and Genomics; P, pathogenic; LP, likely pathogenic; NA, not applicable.

To further assess the pathogenicity of the GH^W112R^ variant, we analyzed interspecific conservation and pathogenicity, comparing GH protein sequences across 12 vertebrate species. **Supplementary Figure 1** illustrates the comparison of GH protein sequences in representative vertebrates, such as *Homo sapiens*, *Bos taurus*, *Ovis aries*, *Sus scrofa*, *Felis catus*, *Equus caballus*, Oryctolagus cuniculus, *Mus musculus*, *Monodelphis domestica*, *Macaca mulatta*, *Anguilla japonica*, and *Oncorhynchus mykiss*. Notably, the Trp112 residue of the GH protein exhibited high conservation across these represented vertebrates (**Supplementary Figure 1C**). The novel missense variant of *GH1*, c.334T>C,p.Trp112Arg, was predicted to be deleterious by seven different predictors (PredictSNP, MMAP, PhD-SNP, polyphen1, polyphen2, SIFT, and SNAP) with confidence scores of 0.87, 0.88, 0.88, 0.74, 0.81, 0.53, and 0.89, respectively. These findings strongly suggest the potential pathogenicity of the c.334T>C,p.Trp112Arg variant in *GH1*.

### 3.4 Homology modeling and structural analysis of human GH

**Figure 3:**
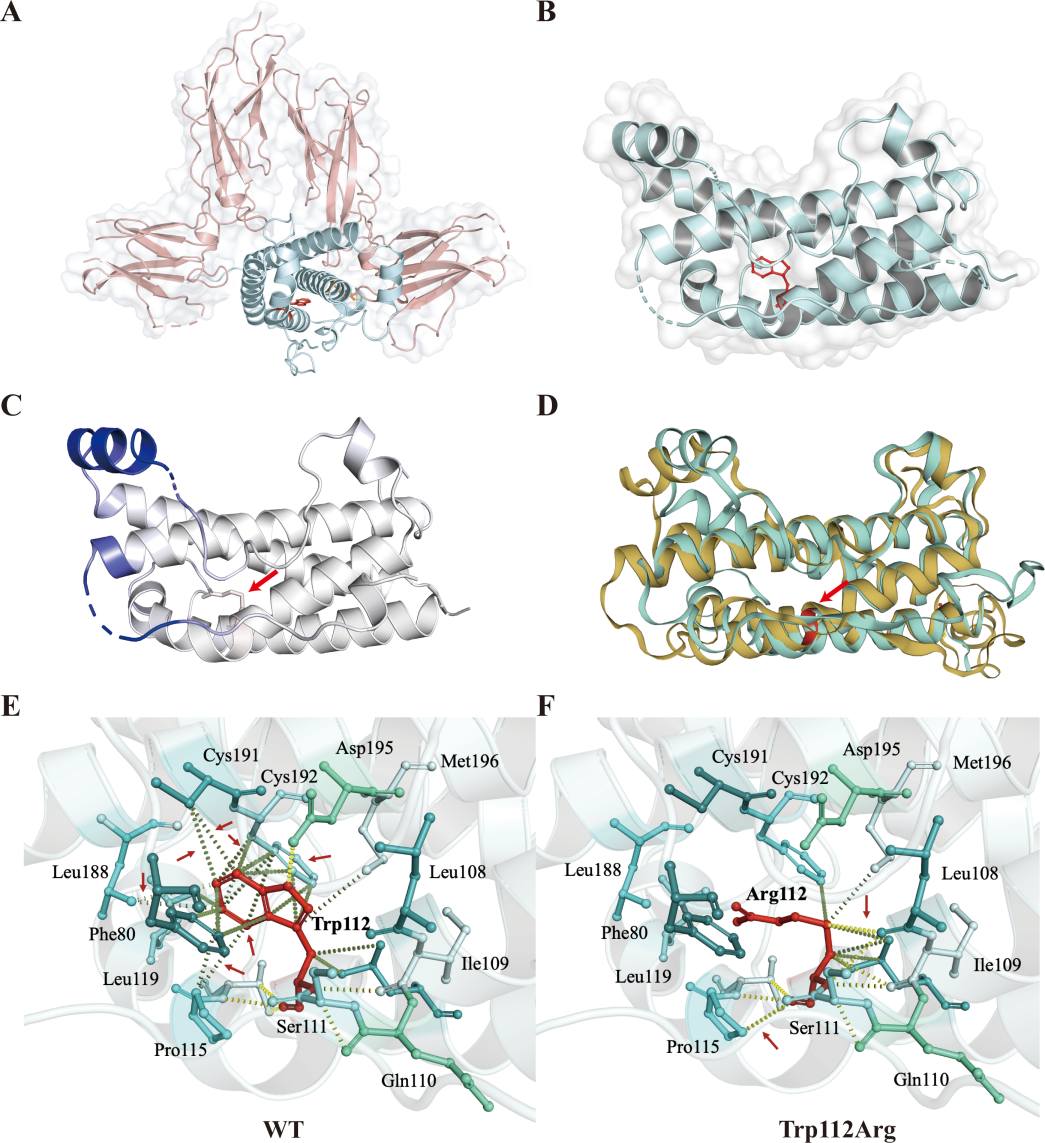
Homology modeling and structural analysis of human growth hormone (GH). **(A, B)** Molecular visualization images for GH and growth hormone receptor (GHR) complexes were produced using PyMOL. The figure shows the relative positions of GH (blue) combined with GHR (pink). Trp112 is represented by a stick model in red. **(C)** Δ Vibrational Entropy EnergyVisual representation of variant: Amino acids are colored based on the vibrational entropy change of the variant. Blue represents a rigidification of structure. The red arrows point to variant at position Trp112Arg. **(D)** Superimposed structural model of wild-type GH (blue-green) and GH^W112R^ (yellow) proteins. The GH^W112R^ variant causes a change in spatial position between adjacent alpha helices. Amino acid residue 112 is shown in red and indicated by an arrow. **(E, F)** Interaction prediction between amino acid residues: Wild-type and variant residues are represented as red sticks. These are placed alongside surrounding residues which are also involved in other types of interactions. Red arrows indicate altered intermolecular forces between amino acids. Hydrogen bonding is represented by a yellow dotted line. Hydrophobic bond interactions are represented as green dotted lines.

In the context of homology modeling and structural analysis of human GH, molecular visualization images for GH and growth hormone receptor (GHR) complexes were generated using PyMOL 2.5 (**Figures 3A, B**). GH comprises four inverse parallel α-helices, and their precise spatial arrangement is crucial for GH-GHR binding. The GH^W112R^ variant was modeled on the I-TASSER online server, revealing the substitution of tryptophan at position 112 with arginine. This substitution significantly reduces the localized flexibility of the α-helix (ΔΔSVib ENCoM: -3.356 kcal·mol^-1^·K^-1^) (**Figure 3C**). Multiple hydrophobic bond interactions were identified between the surrounding amino acid residues (Phe80, Pro115, Leu119, Leu188, Cys191, Cys192) and Trp112, contributing to the stabilization of relative spatial positions between adjacent α-helices (**Figure 3E**). The highly conserved tryptophan was predicted to be buried in the α-helix, and its replacement by charged arginine was expected to disrupt the local helix structure. The GH^W112R^ variant interfered with the local hydrophobic bond of amino acid residue 112 and disrupted the interaction between α-helix II and α-helix IV (**Figures 3D, F**).

### 3.5 Literature review

Including the six children reported in this study, a total of 180 individuals with *GH1* gene variants and IGHD II had been reported. The affected individuals most commonly present with isolated short stature, and some have other clinical manifestations, including a prominent or bossing forehead (n=21/180, 11.7%), depressed nasal bridge (n=12/180, 6.7%), obesity (n=7/180, 3.9%), medial face hypoplasia (n=5/180, 2.8%), small penis (n=4/180, 2.2%), undescended testis (n=3/180, 1.7%), high-pitched voice (n=3/180, 1.7%), and neonatal hypoglycemia (n=3/180, 1.7%). The specific phenotypes of all reported patients with other clinical manifestations besides short stature are listed in **Supplementary Table 1**.

**Figure 4:**
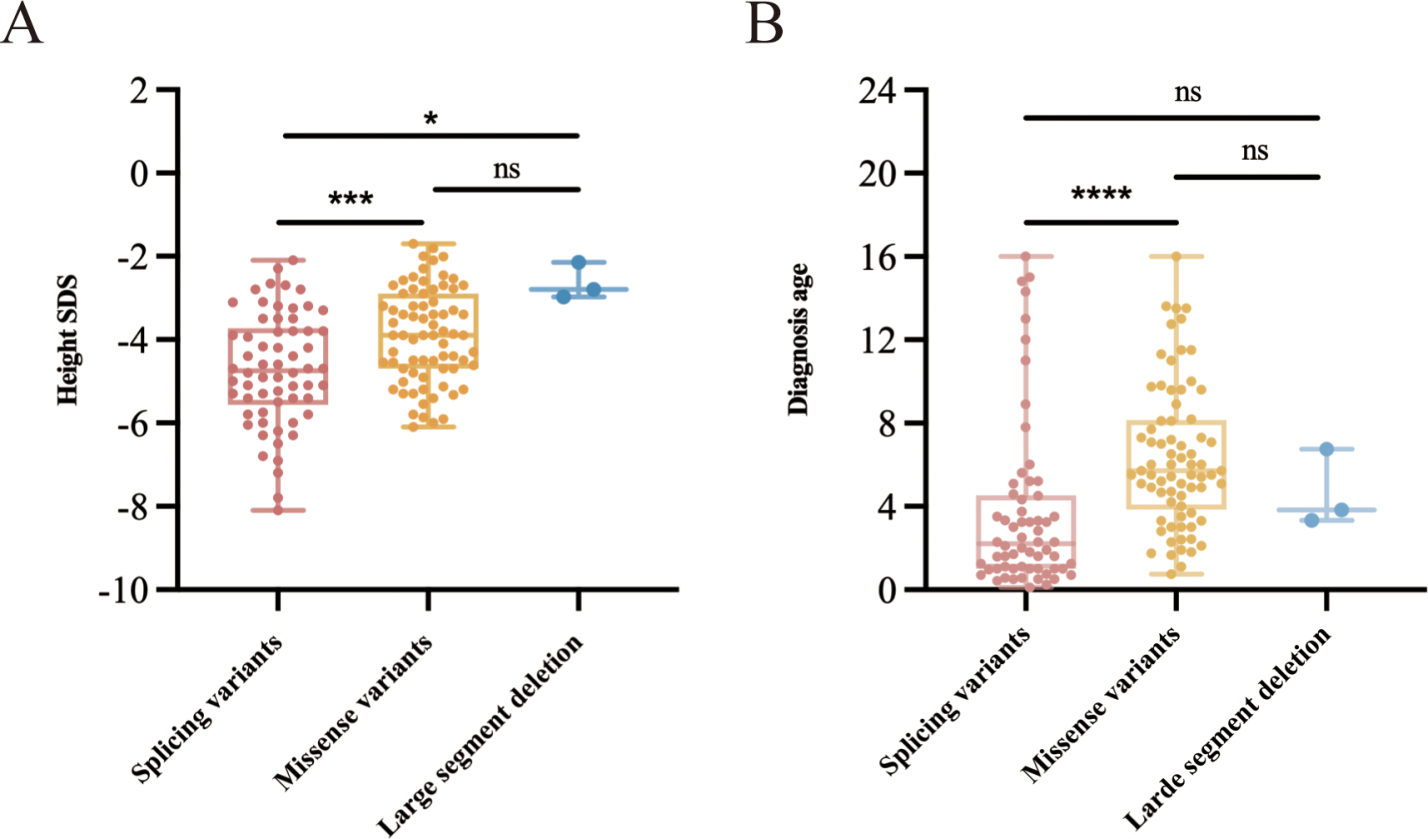
Height SDS and age at diagnosis in children harboring different types of heterozygous variants of the *GH1* gene. **(A)** Comparative analysis of height SDS in children with different *GH1* gene heterozygous variants. **(B)** Comparative analysis of diagnostic age of children with different *GH1* gene heterozygous variants. * indicates *p* < 0.05, *** indicate *p* < 0.001, and **** indicate *p* < 0.0001.

We conducted a comparative analysis of height SDS in children with various heterozygous variants of the *GH1* gene, specifically splice variants (n=62), missense variants (n=73), and large segment deletions (n=3). Following the application of pairwise comparison with Bonferroni-corrected significance levels, it was determined that the height SDS change in patients with *GH1* heterozygous splicing variants was significantly lower compared to patients with *GH1* heterozygous missense variants and large segment deletion (*P*=0.0002 and *P*=0.0104, respectively) (**Figure 4A**). Furthermore, individuals harboring the splice variant exhibited the earliest age of diagnosis, with a median (interquartile range) of 2.2 (1.0-4.3) years, potentially attributable to their severe short stature (**Figure 4B**).

## 4 Discussion

In humans, GH has a variety of physiological and metabolic effects, and its key role in postnatal growth is beyond dispute (18, 19). In the pituitary gland, the *GH1* gene produces a 191 amino acid peptide, arranged antiparallel up-up-down-down with four helices (20). The *GH1* gene, located on chromosome 17q23.3, contains five exons, and four variants were identified in patients 1, 2, 5, and 6, all of which are novel. Among them, one was a missense variant, and the rest were deletions. The c.334T>C, p.Trp112Arg variant described in patient 2 resulted in the replacement of tryptophan with arginine at residue 112 in the GH protein. As shown in **Figure 3**, the unique structural disturbance of Trp112Arg resulted in a significant change in the local structure of the variant, increasing the rigidification of the local protein. Additionally, the GH^W112R^ variant disrupts a large number of hydrophobic bonds around amino acid residue 112, affecting the spatial position between α-helix II and α helix IV, potentially weakening the GH-GHR interaction.

Until now, approximately 124 *GH1* gene variants have been reported in the Human Gene Mutation Database, including missense variants, nonsense variants, segment deletions, shifted code variants, and splice variants. Splicing variation is common in IGHD type II (5), leading to exon 3 skipping and generating a 17.5 kDa protein isoform missing the protein linkage domain between the first two helices of the GH protein and a cysteine residue (C53). This causes the protein to misfold, producing a dominant-negative effect on the wild-type protein (20, 21). In this study, the *GH1* gene splice variant was present in only 2 cases (one-third of the patients), whereas half of the patients had complete or partial heterozygous deletions of the *GH* gene. These large-band deletions, not reported previously, differed from those in IGHD type I, ranging from 6.7kb to 45kb (22). In contrast, the patients in our cohort had smaller deletion fragments, with patient 1 carrying a deletion in exons 2-5 of the *GH1* gene and patients 5 and 6 carrying deletions of 1.5 kb and 1.7 kb, respectively (the deletion fragments contained only the *GH1* gene). Notably, heterozygous deletions of large segments of the *GH1* gene leading to short stature are exceedingly uncommon. To the best of our knowledge, there are no reports of children with short stature due to heterozygous deletion of large segments of the *GH1* gene. In contrast, we reported three out of six IGHD II patients with large fragment heterozygous deletion of the *GH1* gene, which is different from previous knowledge of IGHD II. Classical splicing variants results in skipping of exon 3, which generates a 17.5-kDa peptide. Increasing production of a 17.5-kDa isoform exhibits a dominant-negative effect on the secretion of the 22-kDa isoform both *in vitro* and *in vivo* and in the transgenic animals (23). Furthermore, the accumulation of the 17.5-kDa isoform in cytosol proves toxic to the cells (24). This goes some way to explaining why splicing variants cause a more severe phenotype compared to missense variants.

We conducted a comprehensive review of patients exhibiting short stature as a result of *GH1* gene variants documented in existing literature. The majority of affected individuals exhibit short stature as the primary clinical manifestation, with additional features such as a prominent or bossing forehead, depressed nasal bridge, obesity, medial face hypoplasia, small penis, undescended testis, high-pitched voice, and neonatal hypoglycemia. Clinical severity of IGHD II varies, and it has been shown to correlate with the ratios of GH1 transcripts themselves. The expression of normal GH1 allele transcripts can vary and that the relative amounts of normal and mutant determine severity and penetrance of IGHD II (25). Previous reports have shown that patients with IGHD type II exhibited great variability in their stature, ranging from −4.5SDS to −1.0SDS (25, 26). This study reported six patients exhibiting heterozygous variants of the *GH1* gene, demonstrating a height of -3.95 ± 1.41 SDS and the peak GH value was 2.8 ± 2.2ng/mL at the time of initial assessment. Patient 5 had a height of -2.97 SDS, while her mother did not appear to be very severely height impaired (−1.0SDS). This suggested that the phenotypic heterogeneity could be observed even within a family. In addition to the short stature phenotype, patient 4 also manifested a prominent forehead, undescended testis, and a small penis. In addition, he was found to have a heart murmur on physical examination, and cardiac ultrasound revealed moderate tricuspid valve insufficiency combined with pulmonary hypertension, a previously unreported phenotype. Fofanova et al. reported a case of aortic coarctation in a child with different forms of splicing variation (c.291 + 2T>C) in the *GH1* gene (22). Gregory et al. also reported a case of IGHD with cardiac abnormality (27). The above findings overlap in their assertion that IGHD children may be associated with abnormal cardiac phenotypes. It is highly conceivable that pulmonary hypertension represents a clinical manifestation of IGHD II. Nonetheless, more evidence is warranted to confirm this. The efficacy of rhGH therapy in IGHD II children is indisputable. In our study, IGHD II children treated with rhGH experienced an increase in height by 1.21 ± 0.3 SDS in the first six months of treatment and 1.79 ± 0.15 SDS in the first year. The longer the duration of treatment, the more significant the improvement in the standard deviation score for height. Patient 1 received rhGH replacement therapy after diagnosis (6.9 years) and was above the gene-target height at age 16 years. Through long-term replacement therapy with GH, patient 1 achieved an adult height above his genetic potential, even though he interrupted treatment for 3 years. Patient 2, Patient 3, and Patient 4 were treated for a shorter period of time (0.5 to 3 years) and were not followed up to adult height; therefore, they would need to be treated for a longer period of time to achieve better height improvement.

In summary, this study presents the *GH1* mutation rate among short stature patients in Fujian Province, China, utilizing a substantial and representative cohort. Half of the patients exhibited large fragment deletion mutations in the *GH1* gene, a finding that has not been documented in previous literature. Additionally, we report the first case of IGHD II combined with pulmonary hypertension. Our study enriches the genotype-phenotype spectrum of *GH1* gene variants.

## Data Availability

The datasets presented in this study can be found in online repositories. The names of the repository/repositories and accession number(s) can be found in the article/**Supplementary Material**.

## Supplementary material

Supplementary Figure 1
**(A)** Magnetic resonance image of the pituitary gland in patient 1. Magnetic resonance imaging (MRI) revealed a small pituitary gland and abnormal signals behind the genu of the corpus callosum. **(B)** Magnetic resonance image of the pituitary gland in patient 2. A magnetic resonance image of the pituitary showing a Rathke’s cleft cyst. **(C)** Sequence conservation of mutated amino acids. Arrows point to the positions of the Trp112Arg variant.
