# Supplementary figures and images for: The clinical and genetic aspects of six individuals with GH1 variants and isolated growth hormone deficiency type II
Source: Front Endocrinol (Lausanne). 2024 Oct 7;15:1363050. doi: 10.3389/fendo.2024.1363050 (PMC11491352; doi:10.3389/fendo.2024.1363050)

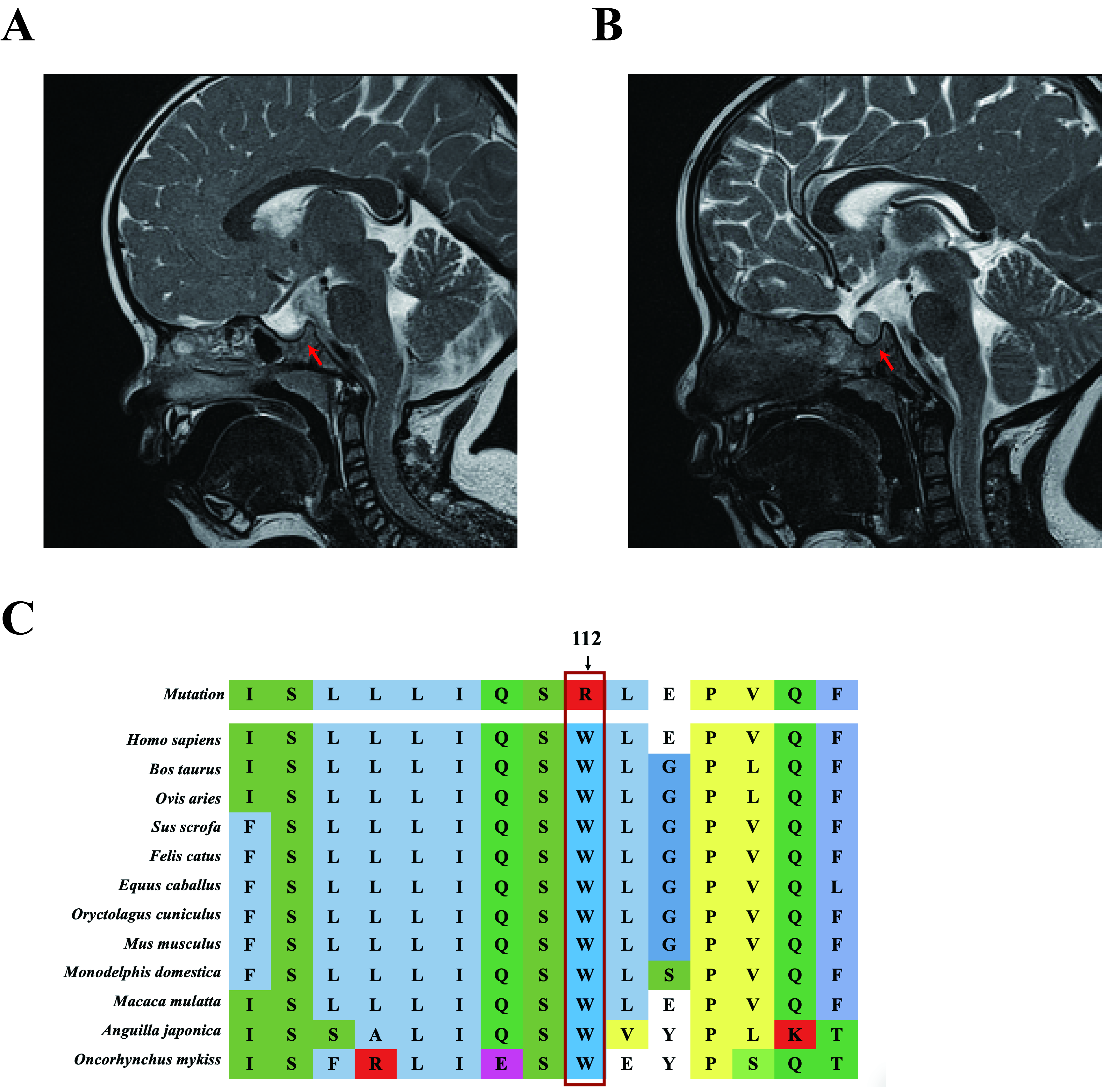

Supplement: Supplementary Figure 1 — (A) Magnetic resonance image of the pituitary gland in patient 1. Magnetic resonance imaging (MRI) revealed a small pituitary gland and abnormal signals behind the genu of the corpus callosum. (B) Magnetic resonance image of the pituitary gland in patient 2. A magnetic resonance image of the pituitary showing a Rathke’s cleft cyst. (C) Sequence conservation of mutated amino acids. Arrows point to the positions of the Trp112Arg variant. [file Image1.tif]
