# Supplementary material for: The clinical and genetic aspects of six individuals with GH1 variants and isolated growth hormone deficiency type II
Source: Front Endocrinol (Lausanne). 2024 Oct 7;15:1363050. doi: 10.3389/fendo.2024.1363050 (PMC11491352; doi:10.3389/fendo.2024.1363050)
Supplement: Supplementary file 2 [file Table1.docx]

**Supplemental table 1** – Clinical manifestations among IGHD typeII caused by GH1 variant

| **Patient** | **Genetics** | **Gender** | **Age at diagnosis** | **HSDS** | **Clinical manifestation** | **reference** |
| --- | --- | --- | --- | --- | --- | --- |
| 1 | c.75T>G | Female | 9.75 | -3.30 | Familial Mediterranean fever | [5] |
| 2 | c.172G>T | Male | 2.30 | -4.10 | Bossing forehead, small nose with a retracted bridge | [14] |
| 3 | c.172G>T | Male | 1.10 | -5.20 | Bossing forehead, small nose with a retracted bridge | [14] |
| 4 | c.199A>T | Male | 8.25 | -4.82 | High-pitched voice, small penis | [6] |
| 5 | c.254C>T | Female | 6.00 | -4.55 | Strabismus of the left eye, learning difficulties | [12] |
| 6 | c.291+1G>A | Male | 1.60 | -4.80 | Central obesity, bossing forehead, hypoglycaemia | [2] |
| 7 | c.291+1G>A | Male | 1.00 | -3.10 | Bossing forehead, saddle nose, blue-like scleras | [4] |
| 8 | c.291+1G>A | Female | 12.00 | -3.20 | Neonatal hypoglycemia, central obesity, medial face hypoplasia, bossing forehead | [9] |
| 9 | c.291+1G>A | Male | 1.00 | -6.30 | Bossing forehead, small penis, cryptorchidism | [13] |
| 10 | c.291+1G>A | Female | 0.70 | -3.50 | Bossing forehead, depressed nasal bridge | [8] |
| 11 | c.291+1G>A | Male | 1.00 | -2.30 | Bossing forehead, depressed nasal bridge | [8] |
| 12 | c.291+1G>A | Female | 2.10 | -5.00 | Bossing forehead, depressed nasal bridge, medial face hypoplasia | [10] |
| 13 | c.291+1G>A | Male | 0.50 | -2.10 | Neonatal jaundice, medial face hypoplasia, bossing forehead, depressed nasal bridge | [10] |
| 14 | c.291+1G>T | Male | 2.30 | -5.40 | Central obesity | [2] |
| 15 | c.291+2T>A | Male | 4.33 | -6.05 | Bossing forehead, pulmonary hypertension, small penis, cryptorchidism | current study |
| 16 | c.291+2T>C | Male | 7.80 | -4.40 | Coarctation of the aorta | [4] |
| 17 | c.291+2T>G | Male | 1.00 | -4.90 | Bossing forehead, medial face hypoplasia | [7] |
| 18 | c.291+5G>A | Female | 0.42 | -3.50 | Neonatal hypoglycemia, central obesity, bossing forehead, medial face hypoplasia | [9] |
| 19 | c.291+6T>C | Male | 14.30 | -7.80 | Hip dysplasia | [1] |
| 20 | c.291+6T>C | Male | 8.90 | -4.20 | High-pitched voice | [1] |
| 21 | c.291+56_77del | Female | 0.58 | -5.80 | Bossing forehead, depressed nasal bridge | [17] |
| 22 | c.307C>T | Male | 4.90 | -6.10 | Bossing forehead, depressed nasal bridge | [15] |
| 23 | c.344C>T | Female | 4.90 | -4.70 | Neonatal hypoglycemia, central obesity, bossing forehead | [2] |
| 24 | c.344C>T | Female | 3.30 | -4.30 | Central obesity, bossing forehead | [2] |
| 25 | c.413A>G | Female | 3.00 | -3.60 | Bossing forehead, saddle nose | [16] |
| 26 | c.456G>A | Female | 4.20 | -5.32 | Bossing forehead, saddle nose, medial face hypoplasia | [3] |
| 27 | c.478C>T | Female | 4.70 | -4.50 | Doll face, bossing forehead, saddle nose, medial face hypoplasia | [10] |
| 28 | c.611G>A | Female | 5.10 | -6.00 | Doll face, medial face hypoplasia, high-pitched voice | [11] |
| 29 | c.626G>A | Male | 5.50 | -3.90 | Central obesity, bossing forehead | [2] |

HSDS: height standard deviation score.

**References**

[1]Cogan JD, Phillips JA, 3rd, Schenkman SS, Milner RD, and Sakati N (1994) Familial growth hormone deficiency: a model of dominant and recessive mutations affecting a monomeric protein. J Clin Endocrinol Metab 79:1261-1265.

[2]de Graaff LC, Argente J, Veenma DC, Herrebout MA, Friesema EC, Uitterlinden AG, Drent ML, Campos-Barros A, and Hokken-Koelega AC (2009) Genetic screening of a Dutch population with isolated GH deficiency (IGHD). Clin Endocrinol (Oxf) 70:742-750.

[3]Fofanova OV, Evgrafov OV, Polyakov AV, Peterkova VA, and Dedov, II (2006) A novel splicing mutation in exon 4 (456G>A) of the GH1 gene in a patient with congenital isolated growth hormone deficiency. Hormones (Athens) 5:288-294.

[4]Fofanova OV, Evgrafov OV, Polyakov AV, Poltaraus AB, Peterkova VA, and Dedov, II (2003) A novel IVS2 -2A>T splicing mutation in the GH-1 gene in familial isolated growth hormone deficiency type II in the spectrum of other splicing mutations in the Russian population. J Clin Endocrinol Metab 88:820-826.

[5]Fritez N, Sobrier ML, Iraqi H, Vié-Luton MP, Netchine I, El Annas A, Pantel J, Collot N, Rose S, Piterboth W, Legendre M, Chraibi A, Amselem S, Kadiri A, and Hilal L (2015) Molecular screening of a large cohort of Moroccan patients with congenital hypopituitarism. Clin Endocrinol (Oxf) 82:876-884.

[6]Gucev Z, Tasic V, Saranac L, Stobbe H, Kratzsch J, Klammt J, and Pfäffle R (2012) A novel GH1 mutation in a family with isolated growth hormone deficiency type II. Horm Res Paediatr 77:200-204.

[7]Kautsar A, Wit JM, and Pulungan A (2019) Isolated Growth Hormone Deficiency Type 2 due to a novel GH1 Mutation: A Case Report. J Clin Res Pediatr Endocrinol 11:426-431.

[8]Kempers MJ, van der Crabben SN, de Vroede M, Alfen-van der Velden J, Netea-Maier RT, Duim RA, Otten BJ, Losekoot M, and Wit JM (2013) Splice site mutations in GH1 detected in previously (Genetically) undiagnosed families with congenital isolated growth hormone deficiency type II. Horm Res Paediatr 80:390-396.

[9]Missarelli C, Herrera L, Mericq V, and Carvallo P (1997) Two different 5' splice site mutations in the growth hormone gene causing autosomal dominant growth hormone deficiency. Hum Genet 101:113-117.

[10]Öztürk AP, Aslanger AD, Bas F, Toksoy G, Karaman V, Bagirova G, Poyrazoglu S, Uyguner ZO, Darendeliler F, and Yavas Abali Z (2024) Phenotype-genotype correlations of GH1 gene variants in patients with isolated growth hormone deficiency (IGHD) or multiple pituitary hormone deficiency (MPHD). Horm Res Paediatr 97(2):126-133.

[11]Petkovic V, Godi M, Pandey AV, Lochmatter D, Buchanan CR, Dattani MT, Eblé A, Flück CE, and Mullis PE (2010) Growth hormone (GH) deficiency type II: a novel GH-1 gene mutation (GH-R178H) affecting secretion and action. J Clin Endocrinol Metab 95:731-739.

[12]Petkovic V, Miletta MC, Boot AM, Losekoot M, Flück CE, Pandey AV, Eblé A, Wit JM, and Mullis PE (2013) Short stature in two siblings heterozygous for a novel bioinactive GH mutant (GH-P59S) suggesting that the mutant also affects secretion of the wild-type GH. Eur J Endocrinol 168:K35-43.

[13]Saitoh H, Fukushima T, Kamoda T, Tanae A, Kamijo T, Yamamoto M, Ogawa M, Hayashi Y, Ohmori S, and Seo H (1999) A Japanese family with autosomal dominant growth hormone deficiency. Eur J Pediatr 158:624-627.

[14]Takahashi I, Takahashi T, Komatsu M, Sato T, and Takada G (2002) An exonic mutation of the GH-1 gene causing familial isolated growth hormone deficiency type II. Clin Genet 61:222-225.

[15]Takahashi Y, Kaji H, Okimura Y, Goji K, Abe H, and Chihara K (1996) Brief report: short stature caused by a mutant growth hormone. N Engl J Med 334:432-436.

[16]Takahashi Y, Shirono H, Arisaka O, Takahashi K, Yagi T, Koga J, Kaji H, Okimura Y, Abe H, Tanaka T, and Chihara K (1997) Biologically inactive growth hormone caused by an amino acid substitution. J Clin Invest 100:1159-1165.

[17]Vivenza D, Guazzarotti L, Godi M, Frasca D, di Natale B, Momigliano-Richiardi P, Bona G, and Giordano M (2006) A novel deletion in the GH1 gene including the IVS3 branch site responsible for autosomal dominant isolated growth hormone deficiency. J Clin Endocrinol Metab 91:980-986.
